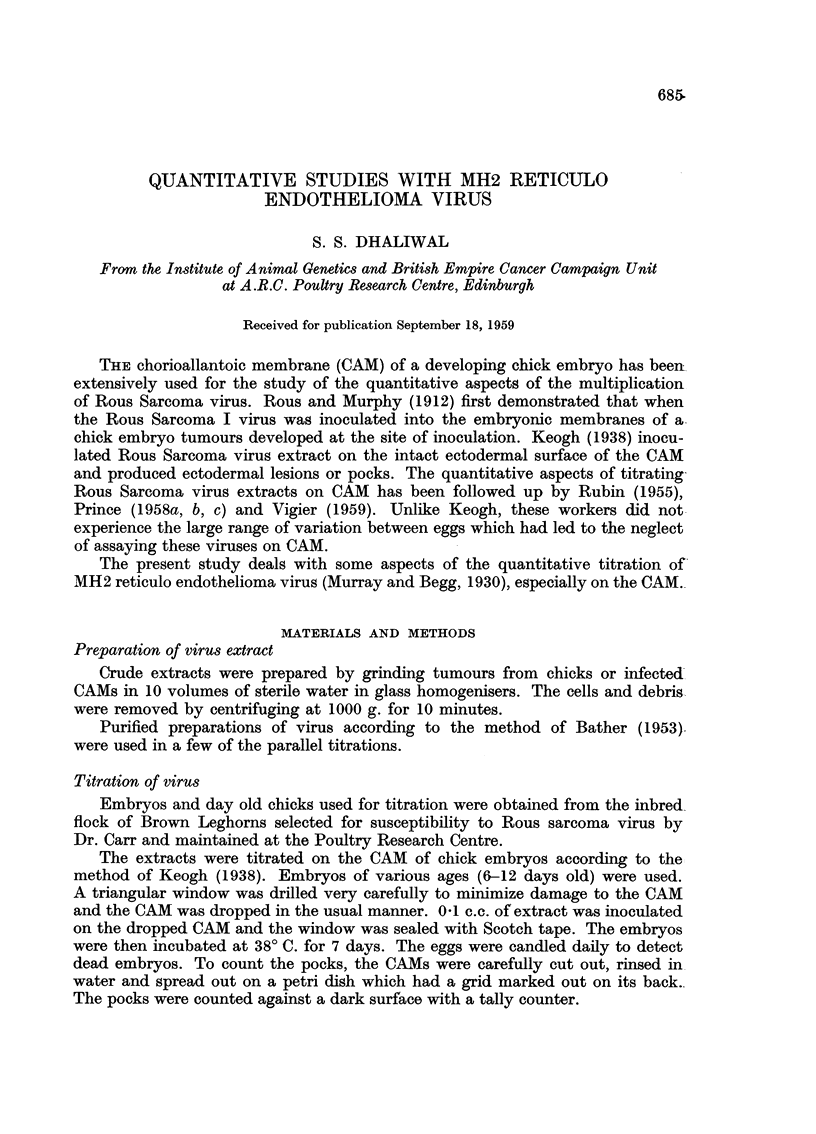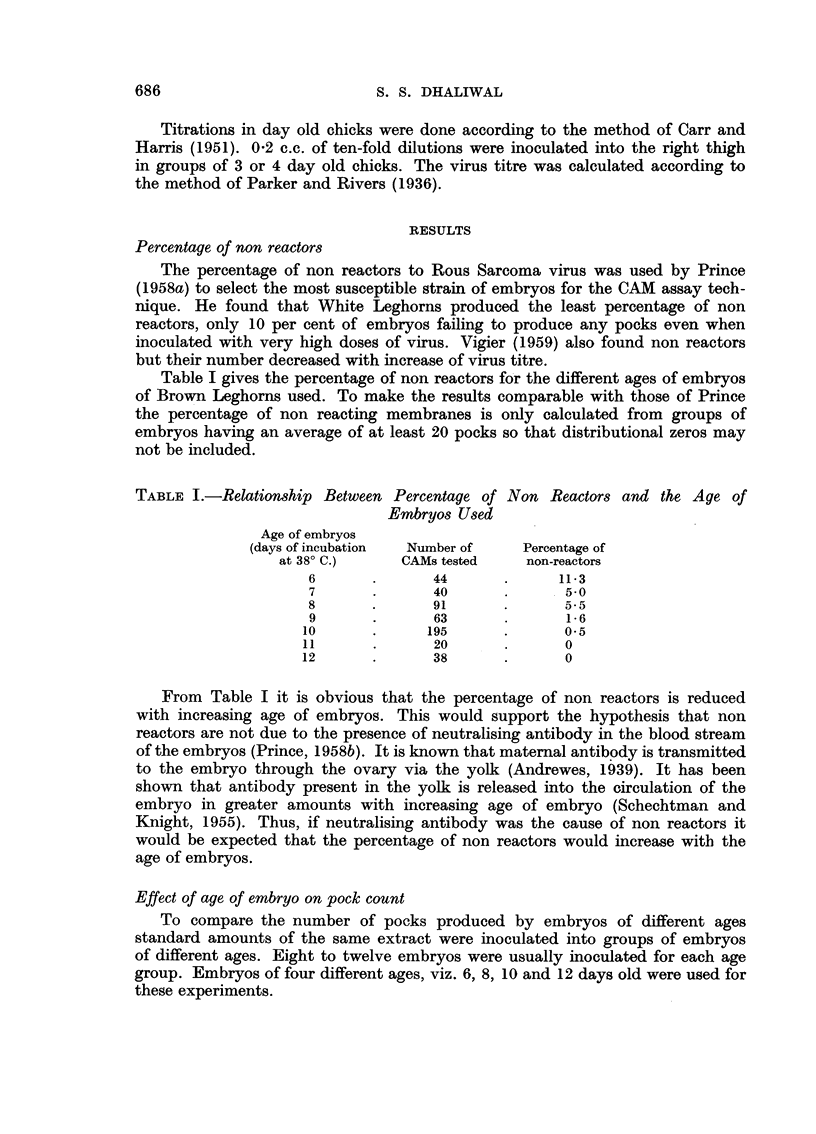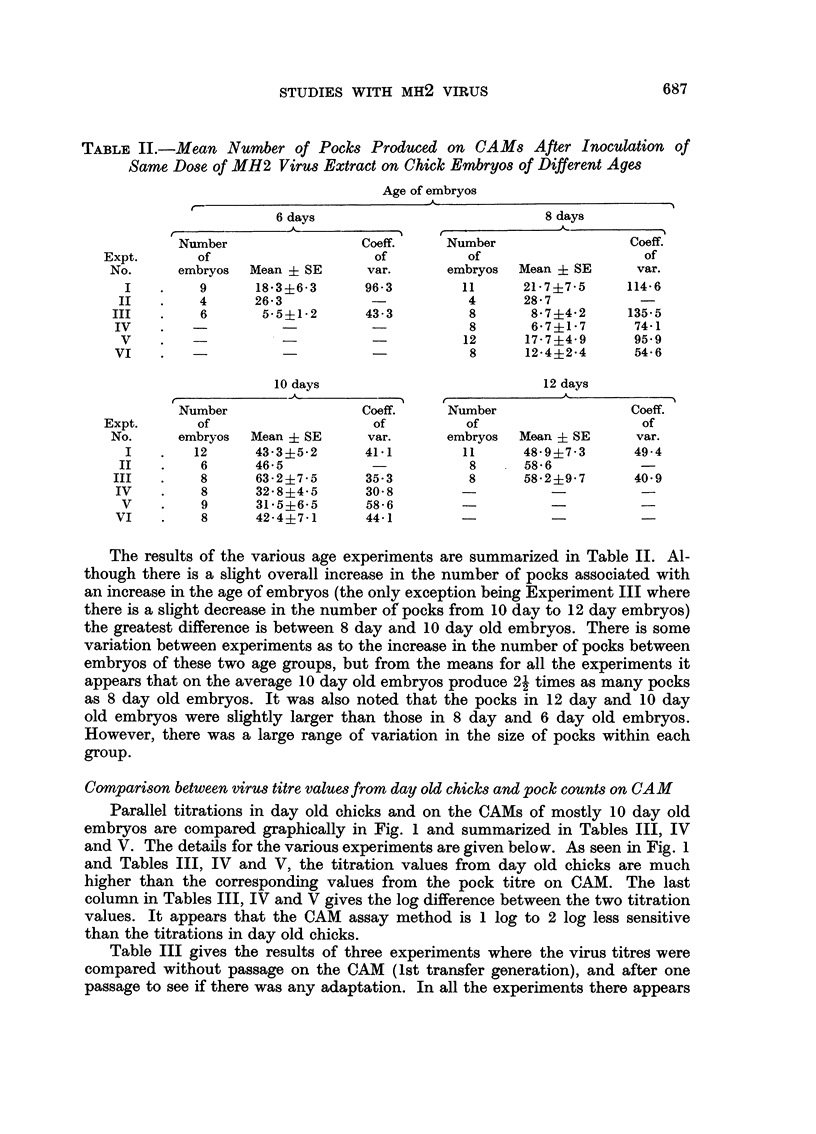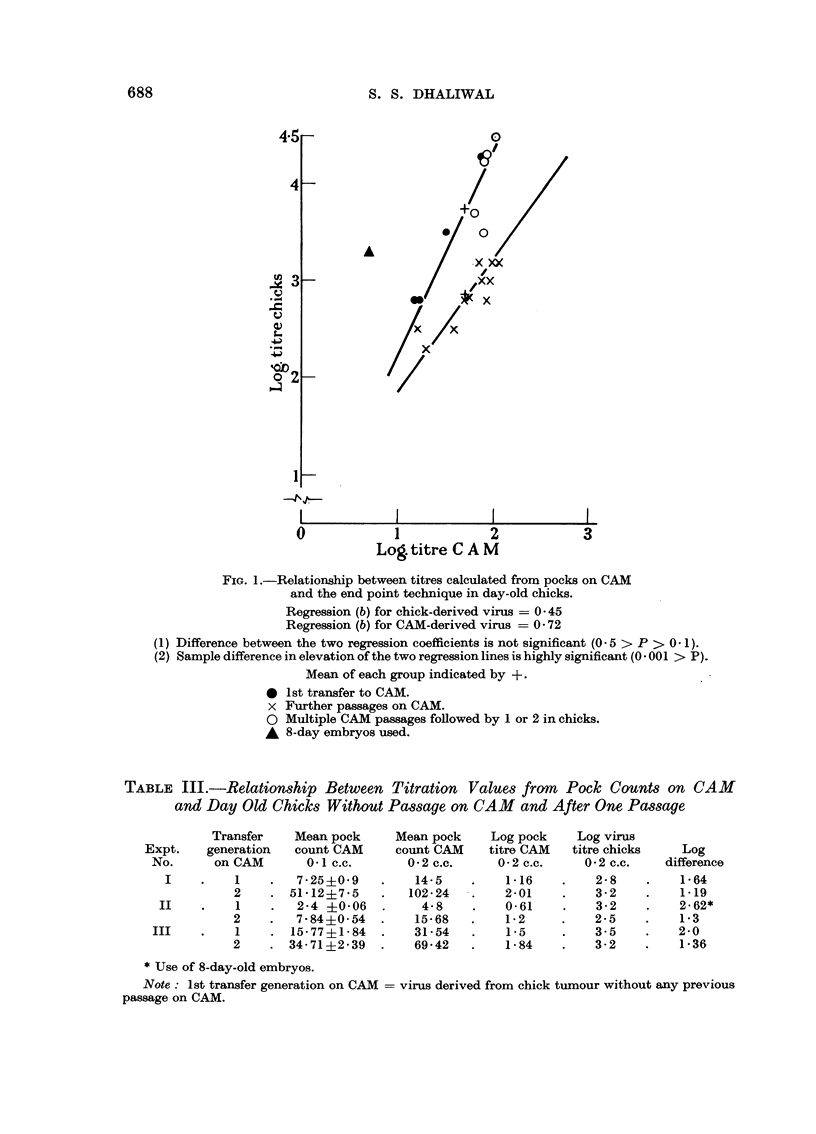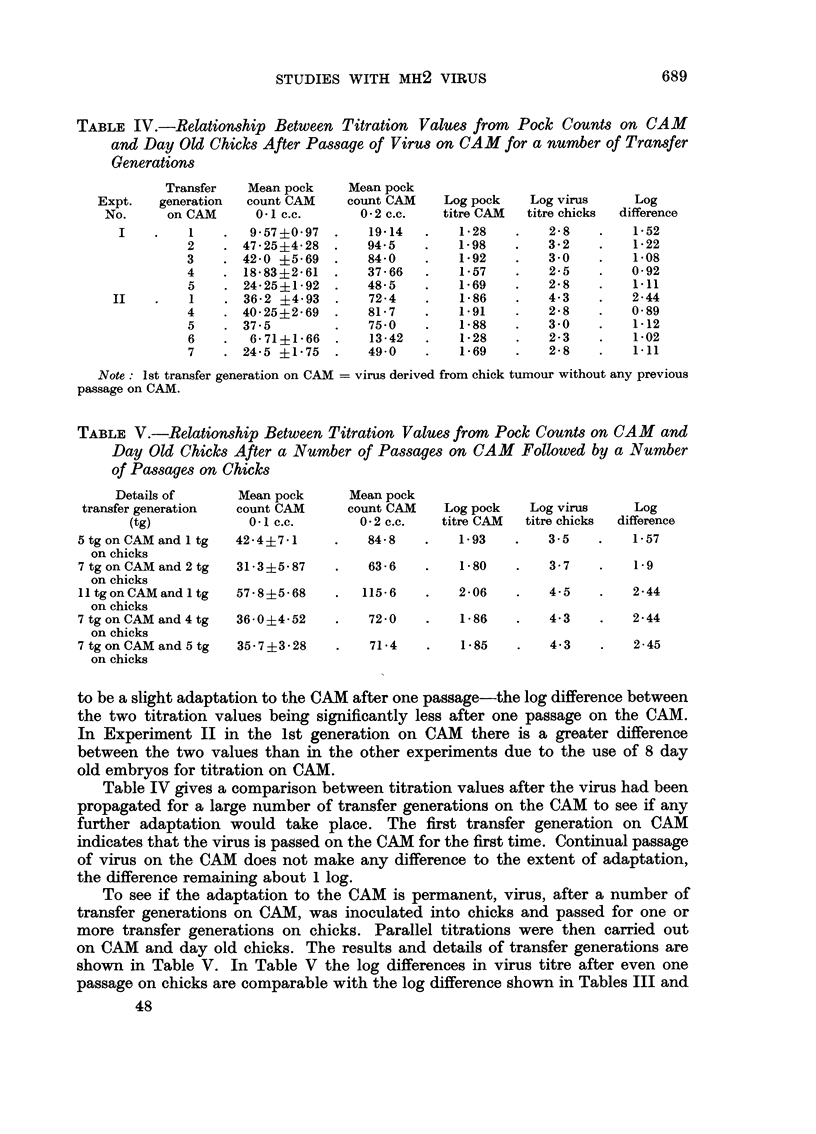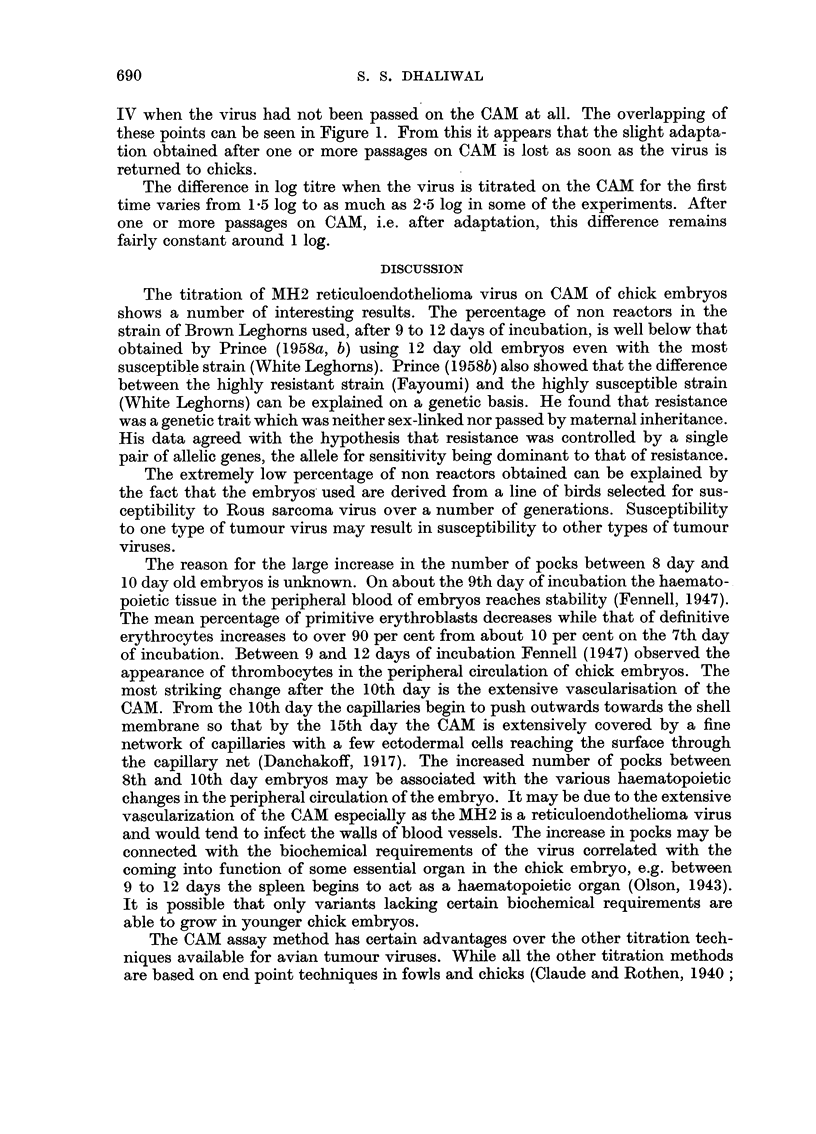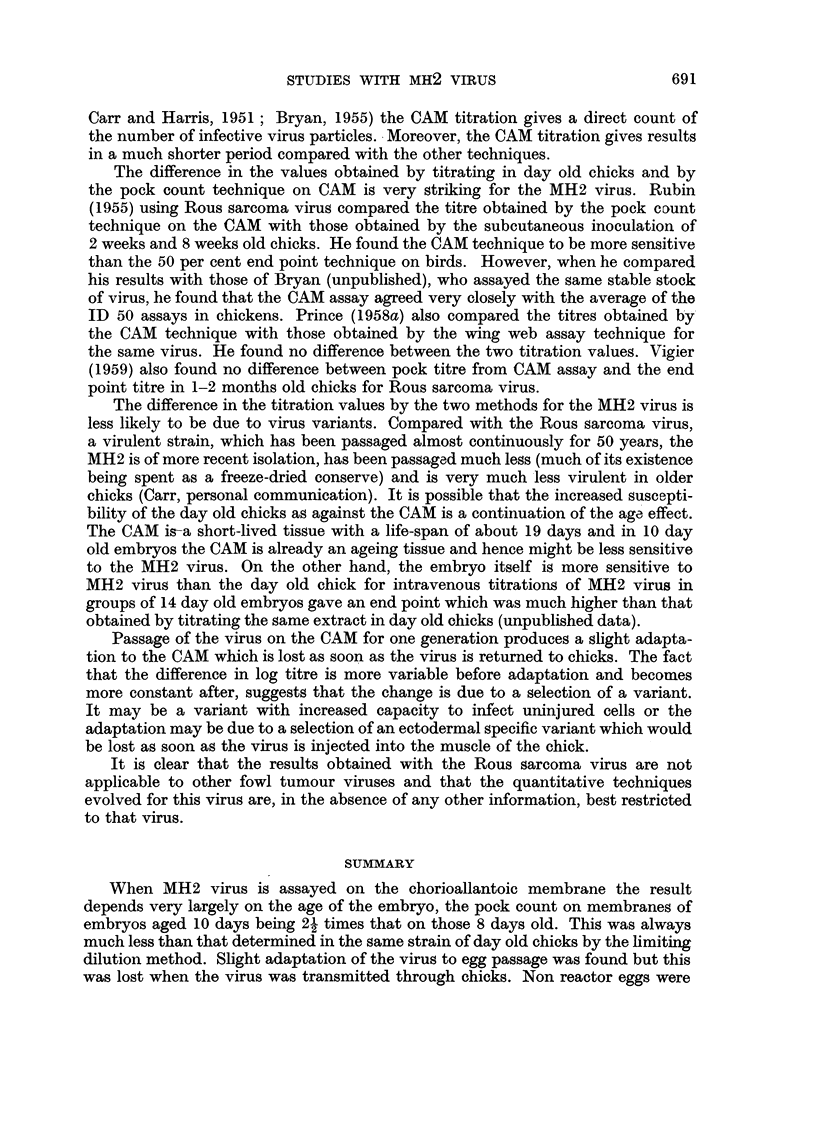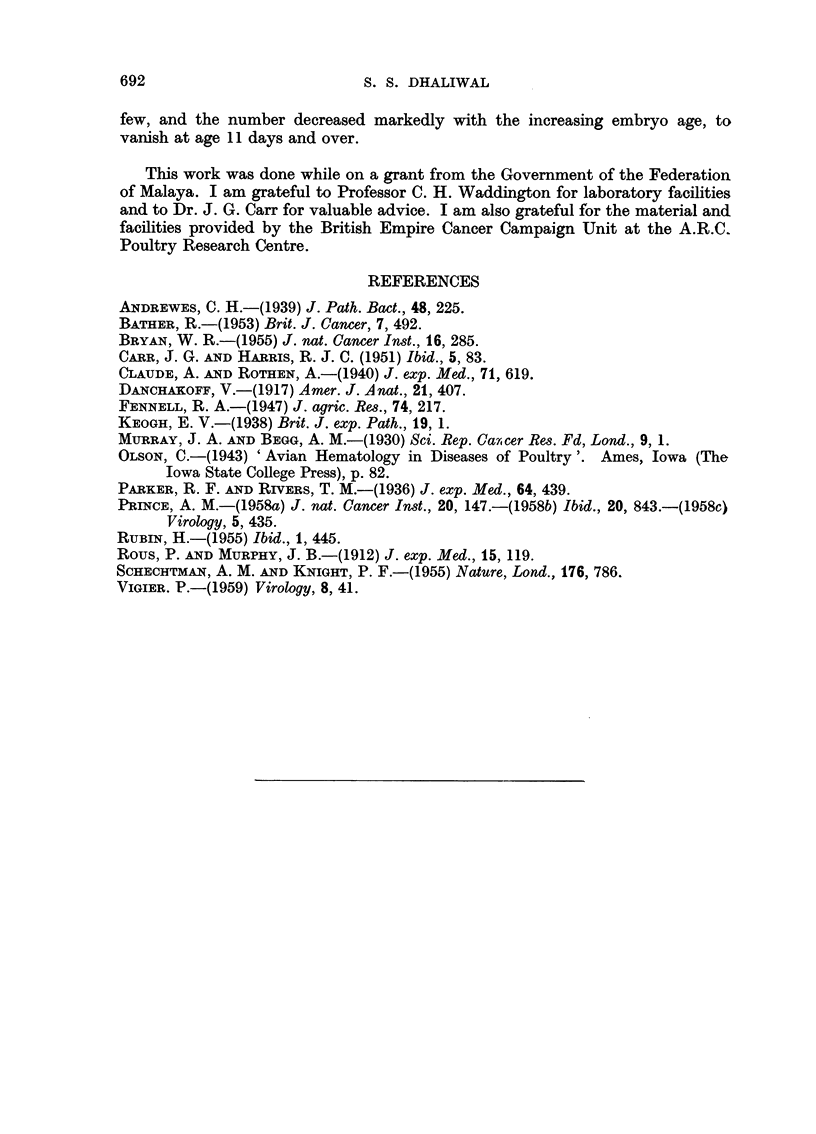# Quantitative Studies with MH2 Reticulo Endothelioma Virus

**DOI:** 10.1038/bjc.1959.76

**Published:** 1959-12

**Authors:** S. S. Dhaliwal


### QUANTITATIVE STUDIES WITH MH2 RETICULO ENDOTHELIOMA VIRUS

S. S. DHALIWAL

*From the Institute of Animal Genetics and British Empire Cancer Campaign Unit at A.R.C. Poultry Research Centre, Edinburgh*

Received for publication September 18, 1959

THE chorioallantoic membrane (CAM) of a developing chick embryo has been extensively used for the study of the quantitative aspects of the multiplication of Rous Sarcoma virus. Rous and Murphy (1912) first demonstrated that when the Rous Sarcoma I virus was inoculated into the embryonic membranes of a chick embryo tumours developed at the site of inoculation. Keogh (1938) inoculated Rous Sarcoma virus extract on the intact ectodermal surface of the CAM and produced ectodermal lesions or pocks. The quantitative aspects of titrating Rous Sarcoma virus extracts on CAM has been followed up by Rubin (1955), Prince (1958*a*, *b*, *c*) and Vigier (1959). Unlike Keogh, these workers did not experience the large range of variation between eggs which had led to the neglect of assaying these viruses on CAM.

The present study deals with some aspects of the quantitative titration of MH2 reticulo endothelioma virus (Murray and Begg, 1930), especially on the CAM.

### MATERIALS AND METHODS

### *Preparation of virus extract*

Crude extracts were prepared by grinding tumours from chicks or infected CAMs in 10 volumes of sterile water in glass homogenisers. The cells and debris were removed by centrifuging at 1000 g. for 10 minutes.

Purified preparations of virus according to the method of Bather (1953) were used in a few of the parallel titrations.

### *Titration of virus*

Embryos and day old chicks used for titration were obtained from the inbred flock of Brown Leghorns selected for susceptibility to Rous sarcoma virus by Dr. Carr and maintained at the Poultry Research Centre.

The extracts were titrated on the CAM of chick embryos according to the method of Keogh (1938). Embryos of various ages (6–12 days old) were used. A triangular window was drilled very carefully to minimize damage to the CAM and the CAM was dropped in the usual manner. 0·1 c.c. of extract was inoculated on the dropped CAM and the window was sealed with Scotch tape. The embryos were then incubated at 38° C. for 7 days. The eggs were candled daily to detect dead embryos. To count the pocks, the CAMs were carefully cut out, rinsed in water and spread out on a petri dish which had a grid marked out on its back. The pocks were counted against a dark surface with a tally counter.

Titrations in day old chicks were done according to the method of Carr and Harris (1951). 0·2 c.c. of ten-fold dilutions were inoculated into the right thigh in groups of 3 or 4 day old chicks. The virus titre was calculated according to the method of Parker and Rivers (1936).

### RESULTS

*Percentage of non reactors*

The percentage of non reactors to Rous Sarcoma virus was used by Prince (1958*a*) to select the most susceptible strain of embryos for the CAM assay technique. He found that White Leghorns produced the least percentage of non reactors, only 10 per cent of embryos failing to produce any pocks even when inoculated with very high doses of virus. Vigier (1959) also found non reactors but their number decreased with increase of virus titre.

Table I gives the percentage of non reactors for the different ages of embryos of Brown Leghorns used. To make the results comparable with those of Prince the percentage of non reacting membranes is only calculated from groups of embryos having an average of at least 20 pocks so that distributional zeros may not be included.

TABLE I.—*Relationship Between Percentage of Non Reactors and the Age of Embryos Used*

| Age of embryos (days of incubation at 38° C.) | Number of CAMs tested | Percentage of non-reactors |
|---|---|---|
| 6 | 44 | 11·3 |
| 7 | 40 | 5·0 |
| 8 | 91 | 5·5 |
| 9 | 63 | 1·6 |
| 10 | 195 | 0·5 |
| 11 | 20 | 0 |
| 12 | 38 | 0 |

From Table I it is obvious that the percentage of non reactors is reduced with increasing age of embryos. This would support the hypothesis that non reactors are not due to the presence of neutralising antibody in the blood stream of the embryos (Prince, 1958*b*). It is known that maternal antibody is transmitted to the embryo through the ovary via the yolk (Andrewes, 1939). It has been shown that antibody present in the yolk is released into the circulation of the embryo in greater amounts with increasing age of embryo (Schechtman and Knight, 1955). Thus, if neutralising antibody was the cause of non reactors it would be expected that the percentage of non reactors would increase with the age of embryos.

*Effect of age of embryo on pock count*

To compare the number of pocks produced by embryos of different ages standard amounts of the same extract were inoculated into groups of embryos of different ages. Eight to twelve embryos were usually inoculated for each age group. Embryos of four different ages, viz. 6, 8, 10 and 12 days old were used for these experiments.

TABLE II.—*Mean Number of Pocks Produced on CAMs After Inoculation of Same Dose of MH2 Virus Extract on Chick Embryos of Different Ages*

| | Age of embryos | | | | | |
|---|---|---|---|---|---|---|
| | 6 days | | | 8 days | | |
| Expt. No. | Number of embryos | Mean ± SE | Coeff. of var. | Number of embryos | Mean ± SE | Coeff. of var. |
| I | 9 | 18·3±6·3 | 96·3 | 11 | 21·7±7·5 | 114·6 |
| II | 4 | 26·3 | — | 4 | 28·7 | — |
| III | 6 | 5·5±1·2 | 43·3 | 8 | 8·7±4·2 | 135·5 |
| IV | — | — | — | 8 | 6·7±1·7 | 74·1 |
| V | — | — | — | 12 | 17·7±4·9 | 95·9 |
| VI | — | — | — | 8 | 12·4±2·4 | 54·6 |

| | 10 days | | | 12 days | | |
|---|---|---|---|---|---|---|
| Expt. No. | Number of embryos | Mean ± SE | Coeff. of var. | Number of embryos | Mean ± SE | Coeff. of var. |
| I | 12 | 43·3±5·2 | 41·1 | 11 | 48·9±7·3 | 49·4 |
| II | 6 | 46·5 | — | 8 | 58·6 | — |
| III | 8 | 63·2±7·5 | 35·3 | 8 | 58·2±9·7 | 40·9 |
| IV | 8 | 32·8±4·5 | 30·8 | — | — | — |
| V | 9 | 31·5±6·5 | 58·6 | — | — | — |
| VI | 8 | 42·4±7·1 | 44·1 | — | — | — |

The results of the various age experiments are summarized in Table II. Although there is a slight overall increase in the number of pocks associated with an increase in the age of embryos (the only exception being Experiment III where there is a slight decrease in the number of pocks from 10 day to 12 day embryos) the greatest difference is between 8 day and 10 day old embryos. There is some variation between experiments as to the increase in the number of pocks between embryos of these two age groups, but from the means for all the experiments it appears that on the average 10 day old embryos produce 2½ times as many pocks as 8 day old embryos. It was also noted that the pocks in 12 day and 10 day old embryos were slightly larger than those in 8 day and 6 day old embryos. However, there was a large range of variation in the size of pocks within each group.

*Comparison between virus titre values from day old chicks and pock counts on CAM*

Parallel titrations in day old chicks and on the CAMs of mostly 10 day old embryos are compared graphically in Fig. 1 and summarized in Tables III, IV and V. The details for the various experiments are given below. As seen in Fig. 1 and Tables III, IV and V, the titration values from day old chicks are much higher than the corresponding values from the pock titre on CAM. The last column in Tables III, IV and V gives the log difference between the two titration values. It appears that the CAM assay method is 1 log to 2 log less sensitive than the titrations in day old chicks.

Table III gives the results of three experiments where the virus titres were compared without passage on the CAM (1st transfer generation), and after one passage to see if there was any adaptation. In all the experiments there appears

FIG. 1.—Relationship between titres calculated from pocks on CAM and the end point technique in day-old chicks.

Regression ($b$) for chick-derived virus = 0·45
Regression ($b$) for CAM-derived virus = 0·72

(1) Difference between the two regression coefficients is not significant ($0\cdot5 > P > 0\cdot1$).
(2) Sample difference in elevation of the two regression lines is highly significant ($0\cdot001 > P$).

Mean of each group indicated by +.

● 1st transfer to CAM.

× Further passages on CAM.

○ Multiple CAM passages followed by 1 or 2 in chicks.

▲ 8-day embryos used.

TABLE III.—*Relationship Between Titration Values from Pock Counts on CAM and Day Old Chicks Without Passage on CAM and After One Passage*

| Expt. No. | Transfer generation on CAM | Mean pock count CAM 0·1 c.c. | Mean pock count CAM 0·2 c.c. | Log pock titre CAM 0·2 c.c. | Log virus titre chicks 0·2 c.c. | Log difference |
|---|---|---|---|---|---|---|
| I | 1 | 7·25±0·9 | 14·5 | 1·16 | 2·8 | 1·64 |
| | 2 | 51·12±7·5 | 102·24 | 2·01 | 3·2 | 1·19 |
| II | 1 | 2·4 ±0·06 | 4·8 | 0·61 | 3·2 | 2·62* |
| | 2 | 7·84±0·54 | 15·68 | 1·2 | 2·5 | 1·3 |
| III | 1 | 15·77±1·84 | 31·54 | 1·5 | 3·5 | 2·0 |
| | 2 | 34·71±2·39 | 69·42 | 1·84 | 3·2 | 1·36 |

* Use of 8-day-old embryos.

*Note :* 1st transfer generation on CAM = virus derived from chick tumour without any previous passage on CAM.

TABLE IV.—*Relationship Between Titration Values from Pock Counts on CAM and Day Old Chicks After Passage of Virus on CAM for a number of Transfer Generations*

| Expt. No. | Transfer generation on CAM | Mean pock count CAM 0·1 c.c. | Mean pock count CAM 0·2 c.c. | Log pock titre CAM | Log virus titre chicks | Log difference |
|---|---|---|---|---|---|---|
| I | 1 | 9·57±0·97 | 19·14 | 1·28 | 2·8 | 1·52 |
| | 2 | 47·25±4·28 | 94·5 | 1·98 | 3·2 | 1·22 |
| | 3 | 42·0 ±5·69 | 84·0 | 1·92 | 3·0 | 1·08 |
| | 4 | 18·83±2·61 | 37·66 | 1·57 | 2·5 | 0·92 |
| | 5 | 24·25±1·92 | 48·5 | 1·69 | 2·8 | 1·11 |
| II | 1 | 36·2 ±4·93 | 72·4 | 1·86 | 4·3 | 2·44 |
| | 4 | 40·25±2·69 | 81·7 | 1·91 | 2·8 | 0·89 |
| | 5 | 37·5 | 75·0 | 1·88 | 3·0 | 1·12 |
| | 6 | 6·71±1·66 | 13·42 | 1·28 | 2·3 | 1·02 |
| | 7 | 24·5 ±1·75 | 49·0 | 1·69 | 2·8 | 1·11 |

*Note :* 1st transfer generation on CAM = virus derived from chick tumour without any previous passage on CAM.

TABLE V.—*Relationship Between Titration Values from Pock Counts on CAM and Day Old Chicks After a Number of Passages on CAM Followed by a Number of Passages on Chicks*

| Details of transfer generation (tg) | Mean pock count CAM 0·1 c.c. | Mean pock count CAM 0·2 c.c. | Log pock titre CAM | Log virus titre chicks | Log difference |
|---|---|---|---|---|---|
| 5 tg on CAM and 1 tg on chicks | 42·4±7·1 | 84·8 | 1·93 | 3·5 | 1·57 |
| 7 tg on CAM and 2 tg on chicks | 31·3±5·87 | 63·6 | 1·80 | 3·7 | 1·9 |
| 11 tg on CAM and 1 tg on chicks | 57·8±5·68 | 115·6 | 2·06 | 4·5 | 2·44 |
| 7 tg on CAM and 4 tg on chicks | 36·0±4·52 | 72·0 | 1·86 | 4·3 | 2·44 |
| 7 tg on CAM and 5 tg on chicks | 35·7±3·28 | 71·4 | 1·85 | 4·3 | 2·45 |

to be a slight adaptation to the CAM after one passage—the log difference between the two titration values being significantly less after one passage on the CAM. In Experiment II in the 1st generation on CAM there is a greater difference between the two values than in the other experiments due to the use of 8 day old embryos for titration on CAM.

Table IV gives a comparison between titration values after the virus had been propagated for a large number of transfer generations on the CAM to see if any further adaptation would take place. The first transfer generation on CAM indicates that the virus is passed on the CAM for the first time. Continual passage of virus on the CAM does not make any difference to the extent of adaptation, the difference remaining about 1 log.

To see if the adaptation to the CAM is permanent, virus, after a number of transfer generations on CAM, was inoculated into chicks and passed for one or more transfer generations on chicks. Parallel titrations were then carried out on CAM and day old chicks. The results and details of transfer generations are shown in Table V. In Table V the log differences in virus titre after even one passage on chicks are comparable with the log difference shown in Tables III and

IV when the virus had not been passed on the CAM at all. The overlapping of these points can be seen in Figure 1. From this it appears that the slight adaptation obtained after one or more passages on CAM is lost as soon as the virus is returned to chicks.

The difference in log titre when the virus is titrated on the CAM for the first time varies from 1·5 log to as much as 2·5 log in some of the experiments. After one or more passages on CAM, i.e. after adaptation, this difference remains fairly constant around 1 log.

### DISCUSSION

The titration of MH2 reticuloendothelioma virus on CAM of chick embryos shows a number of interesting results. The percentage of non reactors in the strain of Brown Leghorns used, after 9 to 12 days of incubation, is well below that obtained by Prince (1958*a*, *b*) using 12 day old embryos even with the most susceptible strain (White Leghorns). Prince (1958*b*) also showed that the difference between the highly resistant strain (Fayoumi) and the highly susceptible strain (White Leghorns) can be explained on a genetic basis. He found that resistance was a genetic trait which was neither sex-linked nor passed by maternal inheritance. His data agreed with the hypothesis that resistance was controlled by a single pair of allelic genes, the allele for sensitivity being dominant to that of resistance.

The extremely low percentage of non reactors obtained can be explained by the fact that the embryos used are derived from a line of birds selected for susceptibility to Rous sarcoma virus over a number of generations. Susceptibility to one type of tumour virus may result in susceptibility to other types of tumour viruses.

The reason for the large increase in the number of pocks between 8 day and 10 day old embryos is unknown. On about the 9th day of incubation the haematopoietic tissue in the peripheral blood of embryos reaches stability (Fennell, 1947). The mean percentage of primitive erythroblasts decreases while that of definitive erythrocytes increases to over 90 per cent from about 10 per cent on the 7th day of incubation. Between 9 and 12 days of incubation Fennell (1947) observed the appearance of thrombocytes in the peripheral circulation of chick embryos. The most striking change after the 10th day is the extensive vascularisation of the CAM. From the 10th day the capillaries begin to push outwards towards the shell membrane so that by the 15th day the CAM is extensively covered by a fine network of capillaries with a few ectodermal cells reaching the surface through the capillary net (Danchakoff, 1917). The increased number of pocks between 8th and 10th day embryos may be associated with the various haematopoietic changes in the peripheral circulation of the embryo. It may be due to the extensive vascularization of the CAM especially as the MH2 is a reticuloendothelioma virus and would tend to infect the walls of blood vessels. The increase in pocks may be connected with the biochemical requirements of the virus correlated with the coming into function of some essential organ in the chick embryo, e.g. between 9 to 12 days the spleen begins to act as a haematopoietic organ (Olson, 1943). It is possible that only variants lacking certain biochemical requirements are able to grow in younger chick embryos.

The CAM assay method has certain advantages over the other titration techniques available for avian tumour viruses. While all the other titration methods are based on end point techniques in fowls and chicks (Claude and Rothen, 1940 ;

Carr and Harris, 1951 ; Bryan, 1955) the CAM titration gives a direct count of the number of infective virus particles. Moreover, the CAM titration gives results in a much shorter period compared with the other techniques.

The difference in the values obtained by titrating in day old chicks and by the pock count technique on CAM is very striking for the MH2 virus. Rubin (1955) using Rous sarcoma virus compared the titre obtained by the pock count technique on the CAM with those obtained by the subcutaneous inoculation of 2 weeks and 8 weeks old chicks. He found the CAM technique to be more sensitive than the 50 per cent end point technique on birds. However, when he compared his results with those of Bryan (unpublished), who assayed the same stable stock of virus, he found that the CAM assay agreed very closely with the average of the ID 50 assays in chickens. Prince (1958*a*) also compared the titres obtained by the CAM technique with those obtained by the wing web assay technique for the same virus. He found no difference between the two titration values. Vigier (1959) also found no difference between pock titre from CAM assay and the end point titre in 1–2 months old chicks for Rous sarcoma virus.

The difference in the titration values by the two methods for the MH2 virus is less likely to be due to virus variants. Compared with the Rous sarcoma virus, a virulent strain, which has been passaged almost continuously for 50 years, the MH2 is of more recent isolation, has been passaged much less (much of its existence being spent as a freeze-dried conserve) and is very much less virulent in older chicks (Carr, personal communication). It is possible that the increased susceptibility of the day old chicks as against the CAM is a continuation of the age effect. The CAM is a short-lived tissue with a life-span of about 19 days and in 10 day old embryos the CAM is already an ageing tissue and hence might be less sensitive to the MH2 virus. On the other hand, the embryo itself is more sensitive to MH2 virus than the day old chick for intravenous titrations of MH2 virus in groups of 14 day old embryos gave an end point which was much higher than that obtained by titrating the same extract in day old chicks (unpublished data).

Passage of the virus on the CAM for one generation produces a slight adaptation to the CAM which is lost as soon as the virus is returned to chicks. The fact that the difference in log titre is more variable before adaptation and becomes more constant after, suggests that the change is due to a selection of a variant. It may be a variant with increased capacity to infect uninjured cells or the adaptation may be due to a selection of an ectodermal specific variant which would be lost as soon as the virus is injected into the muscle of the chick.

It is clear that the results obtained with the Rous sarcoma virus are not applicable to other fowl tumour viruses and that the quantitative techniques evolved for this virus are, in the absence of any other information, best restricted to that virus.

### SUMMARY

When MH2 virus is assayed on the chorioallantoic membrane the result depends very largely on the age of the embryo, the pock count on membranes of embryos aged 10 days being $2\frac{1}{2}$ times that on those 8 days old. This was always much less than that determined in the same strain of day old chicks by the limiting dilution method. Slight adaptation of the virus to egg passage was found but this was lost when the virus was transmitted through chicks. Non reactor eggs were few, and the number decreased markedly with the increasing embryo age, to vanish at age 11 days and over.

This work was done while on a grant from the Government of the Federation of Malaya. I am grateful to Professor C. H. Waddington for laboratory facilities and to Dr. J. G. Carr for valuable advice. I am also grateful for the material and facilities provided by the British Empire Cancer Campaign Unit at the A.R.C. Poultry Research Centre.

### REFERENCES

ANDREWES, C. H.—(1939) *J. Path. Bact.*, **48**, 225.
BATHER, R.—(1953) *Brit. J. Cancer*, **7**, 492.
BRYAN, W. R.—(1955) *J. nat. Cancer Inst.*, **16**, 285.
CARR, J. G. AND HARRIS, R. J. C. (1951) *Ibid.*, **5**, 83.
CLAUDE, A. AND ROTHEN, A.—(1940) *J. exp. Med.*, **71**, 619.
DANCHAKOFF, V.—(1917) *Amer. J. Anat.*, **21**, 407.
FENNELL, R. A.—(1947) *J. agric. Res.*, **74**, 217.
KEOGH, E. V.—(1938) *Brit. J. exp. Path.*, **19**, 1.
MURRAY, J. A. AND BEGG, A. M.—(1930) *Sci. Rep. Cancer Res. Fd, Lond.*, **9**, 1.
OLSON, C.—(1943) 'Avian Hematology in Diseases of Poultry'. Ames, Iowa (The Iowa State College Press), p. 82.
PARKER, R. F. AND RIVERS, T. M.—(1936) *J. exp. Med.*, **64**, 439.
PRINCE, A. M.—(1958*a*) *J. nat. Cancer Inst.*, **20**, 147.—(1958*b*) *Ibid.*, **20**, 843.—(1958*c*) *Virology*, **5**, 435.
RUBIN, H.—(1955) *Ibid.*, **1**, 445.
ROUS, P. AND MURPHY, J. B.—(1912) *J. exp. Med.*, **15**, 119.
SCHECHTMAN, A. M. AND KNIGHT, P. F.—(1955) *Nature, Lond.*, **176**, 786.
VIGIER. P.—(1959) *Virology*, **8**, 41.